# Supplementary material for: Selective sulfur dioxide adsorption on crystal defect sites on an isoreticular metal organic framework series
Source: Nat Commun. 2017 Feb 15;8:14457. doi: 10.1038/ncomms14457 (PMC5316851; doi:10.1038/ncomms14457)
Supplement: Supplementary Information — Supplementary Figures, Supplementary Tables, Supplementary Methods, Supplementary Notes and Supplementary References [file ncomms14457-s1.pdf]

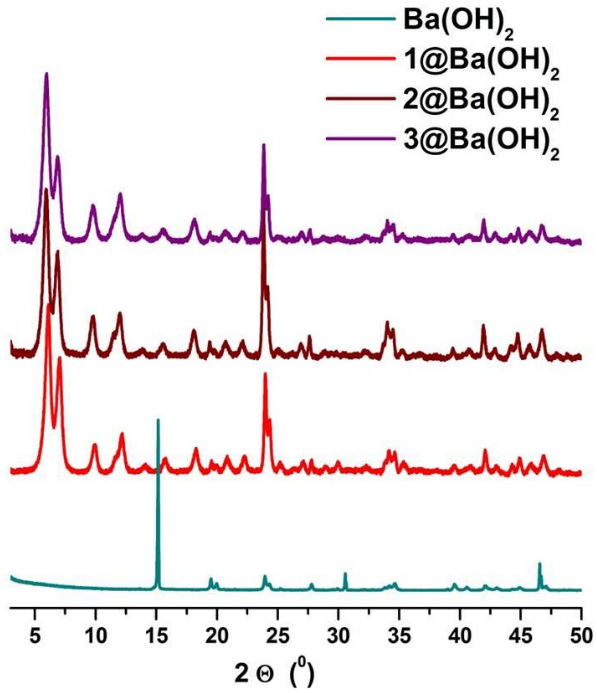

Supplementary Figure 1 / PXRD patterns of **1@Ba(OH)$_2$, 2@Ba(OH)$_2$** and **3@Ba(OH)$_2$** compared with barium hydroxide X-ray pattern

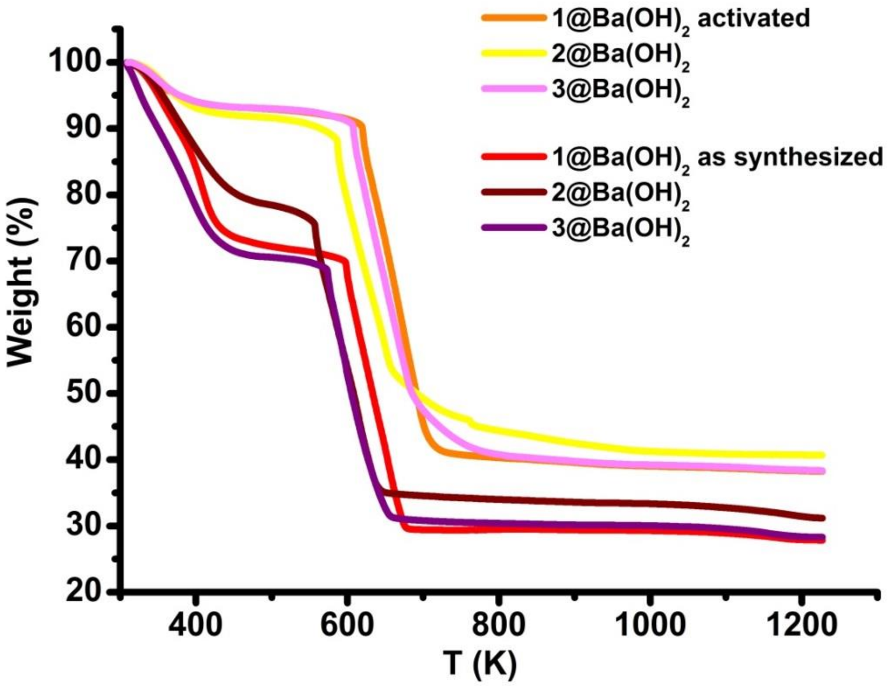

Supplementary Figure 2 / TGA of samples **1@Ba(OH)$_2$, 2@Ba(OH)$_2$** and **3@Ba(OH)$_2$**

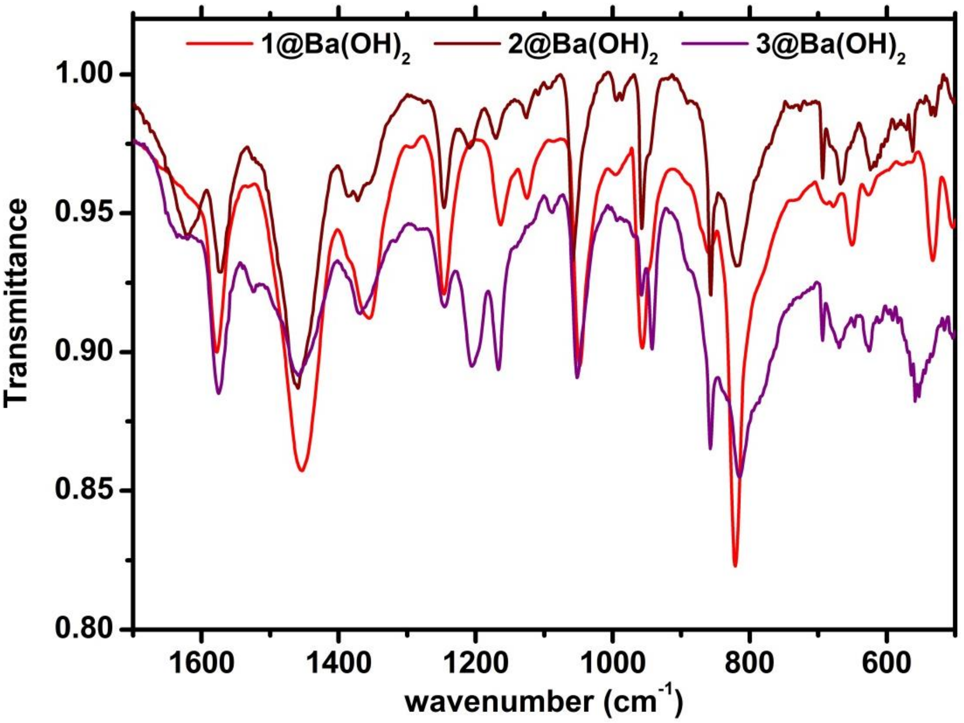

Supplementary Figure 3 / FTIR of samples **1@Ba(OH)₂ , 2@Ba(OH)₂** and **3@Ba(OH)₂** (zoom 1800 - 400cm^{-1})

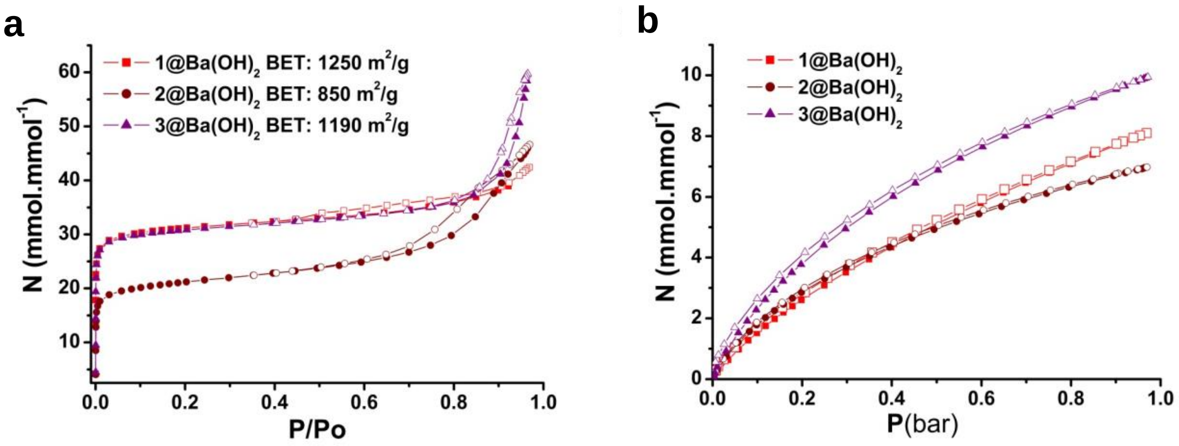

Supplementary Figure 4 / Representation of gravimetric form for the adsorption isotherms of (**a**) N_2 at 77 K an (**b**) CO_2 at 273 K for **1@Ba(OH)₂, 2@Ba(OH)₂** and **3@Ba(OH)₂**. Close symbols indicate adsorption, open symbols indicate desorption

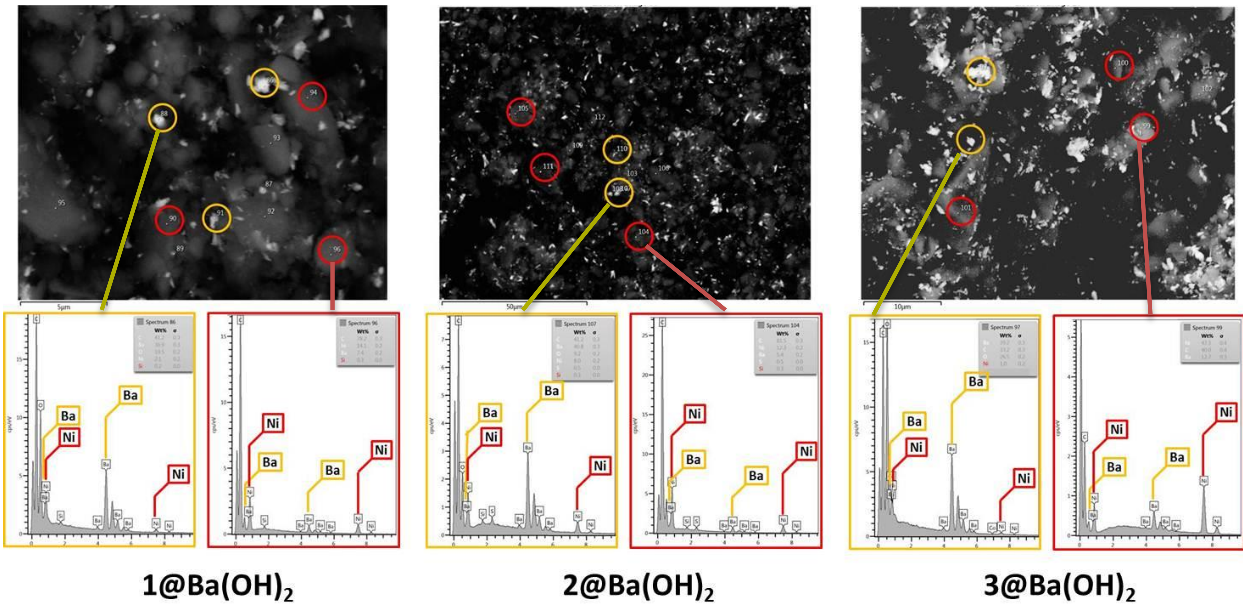

Supplementary Figure 5 / Variable pressure scanning electron microscopy (VP-SEM) and energy dispersive X-ray analyses (EDX) for 1@Ba(OH)2-3@Ba(OH)2 materials. Yellow circles corresponds to Ba rich particles (Ba(OH)2) whereas red circles correspond to Bax[Ni8(OH)3(EtO)3(BDP_X)6-x] MOF particles

a

b

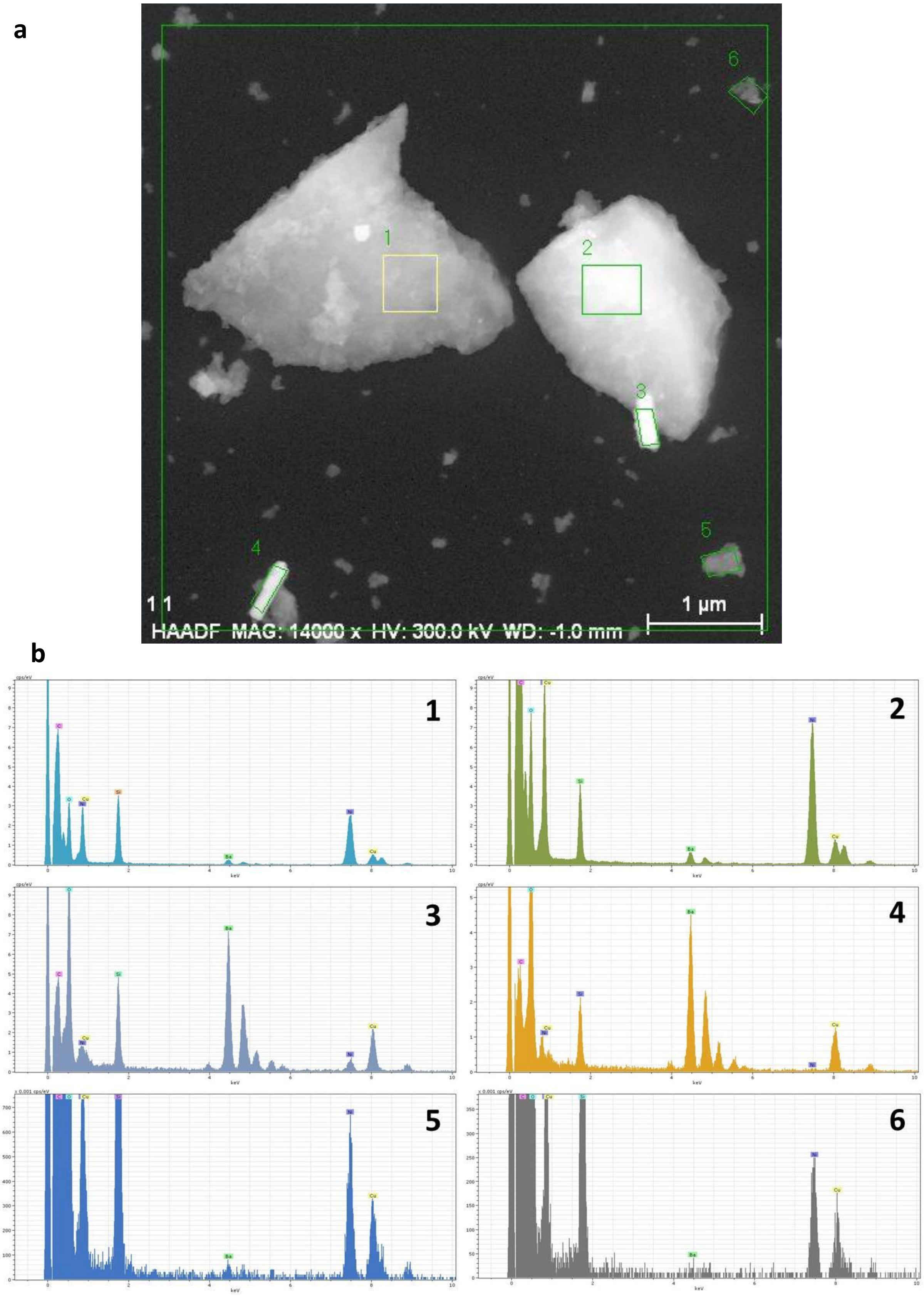

Supplementary Figure 6 / Energy dispersive X-ray spectroscopy analysis of sample **1@Ba(OH)$_2$** by HRTEM, (**a**) HAADF images with analysis area marks, (**b**) EDX-spectra of each area marked

1@Ba(OH)$_2$

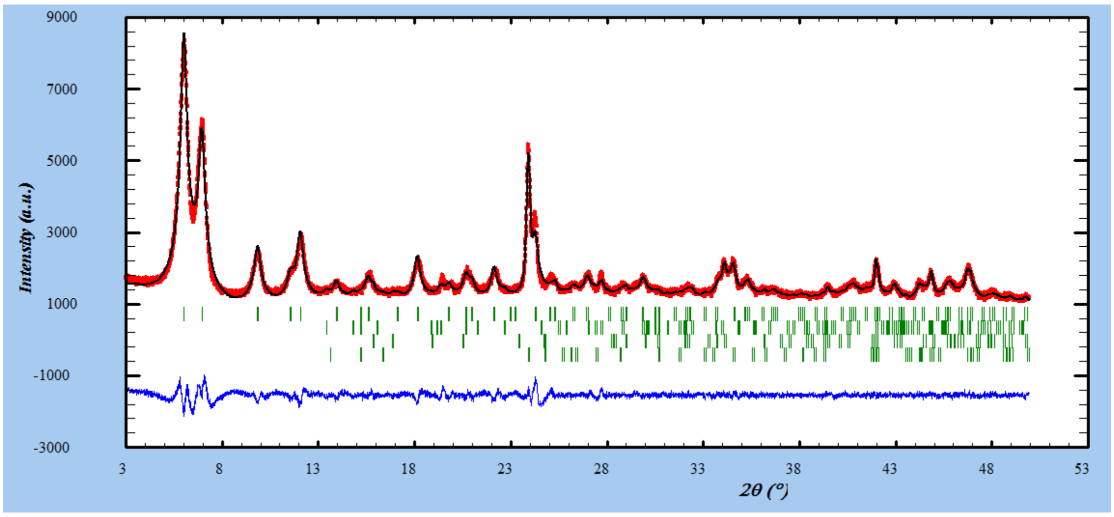

2@Ba(OH)$_2$

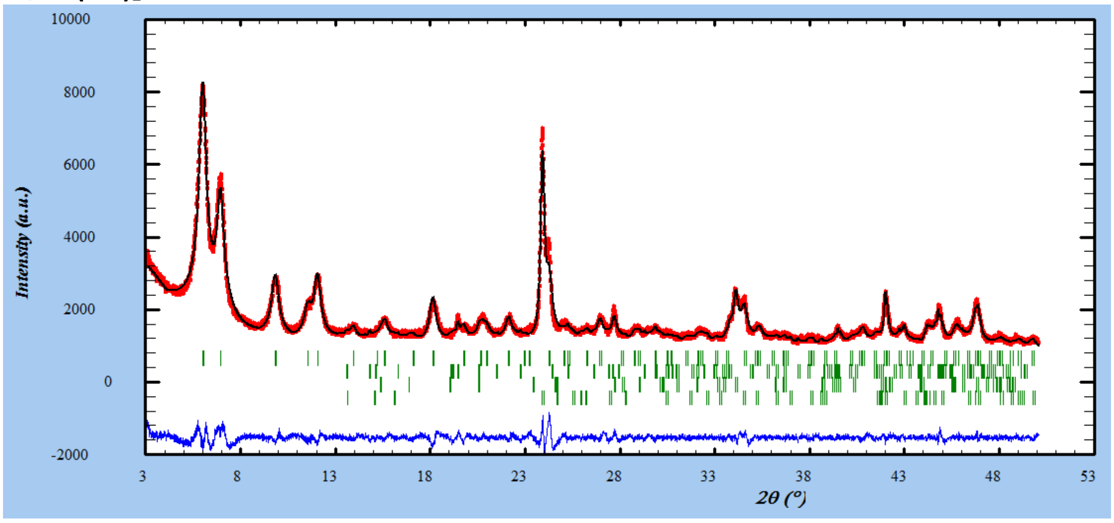

3@Ba(OH)$_2$

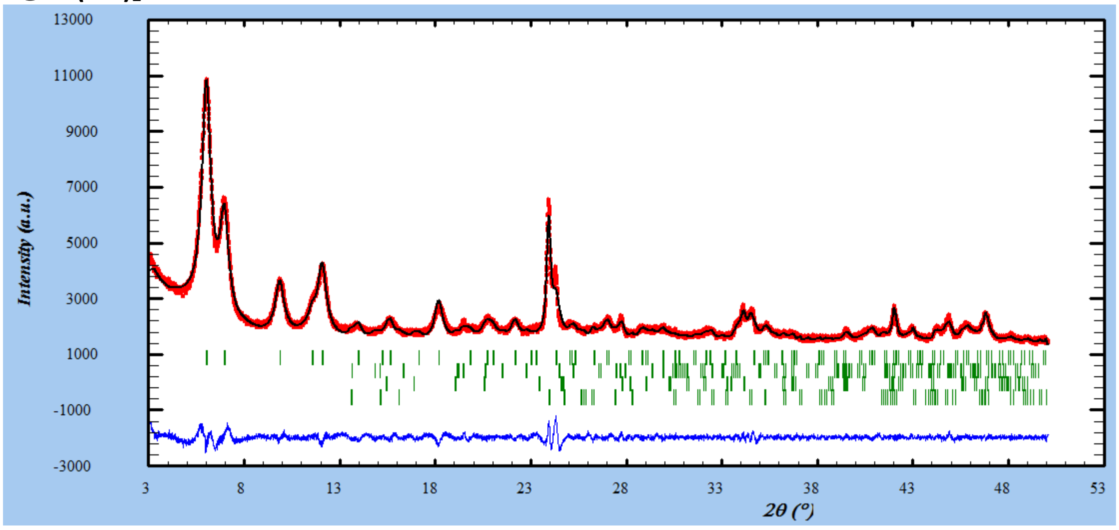

1@KOH

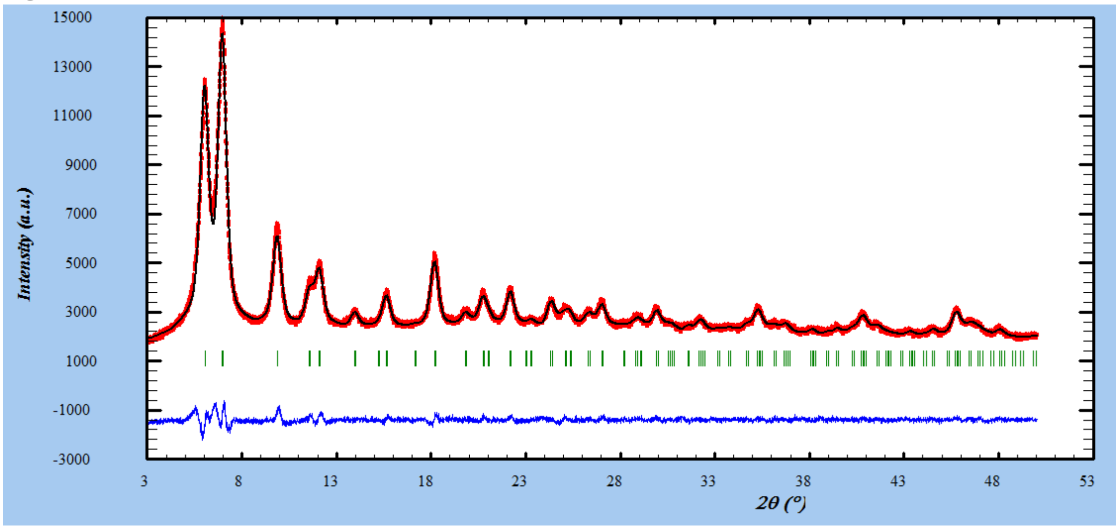

2@KOH

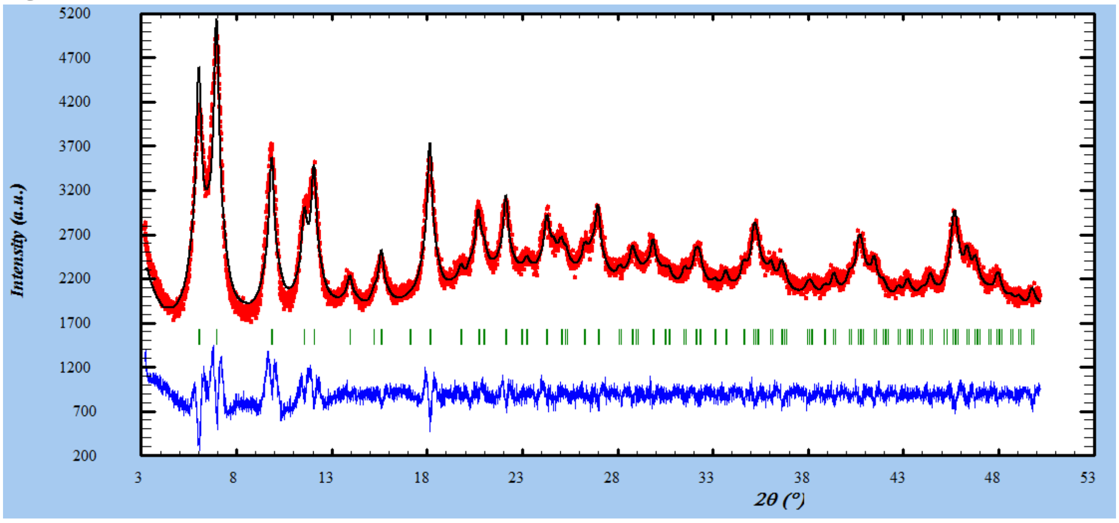

3@KOH

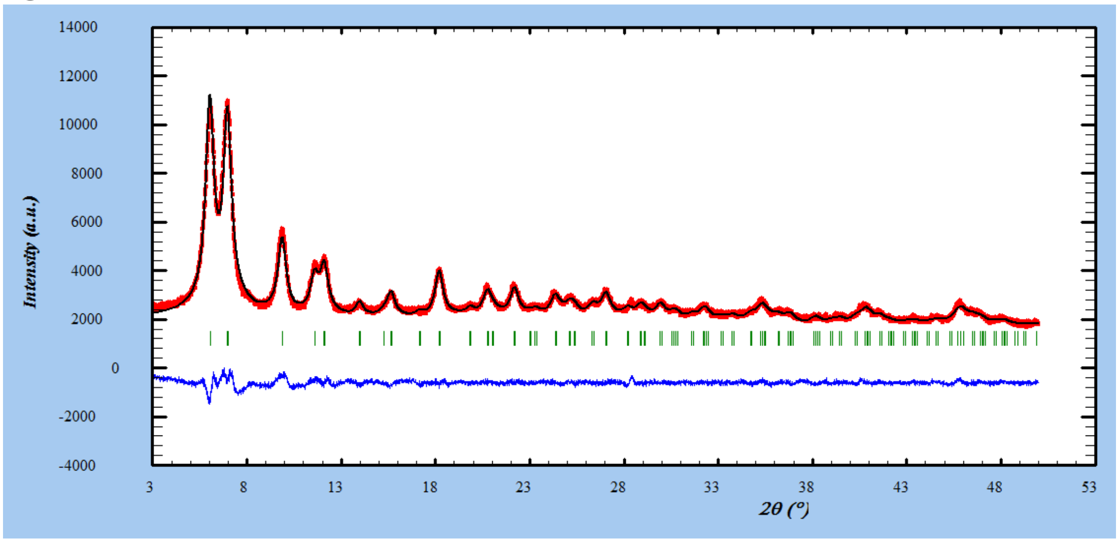

1

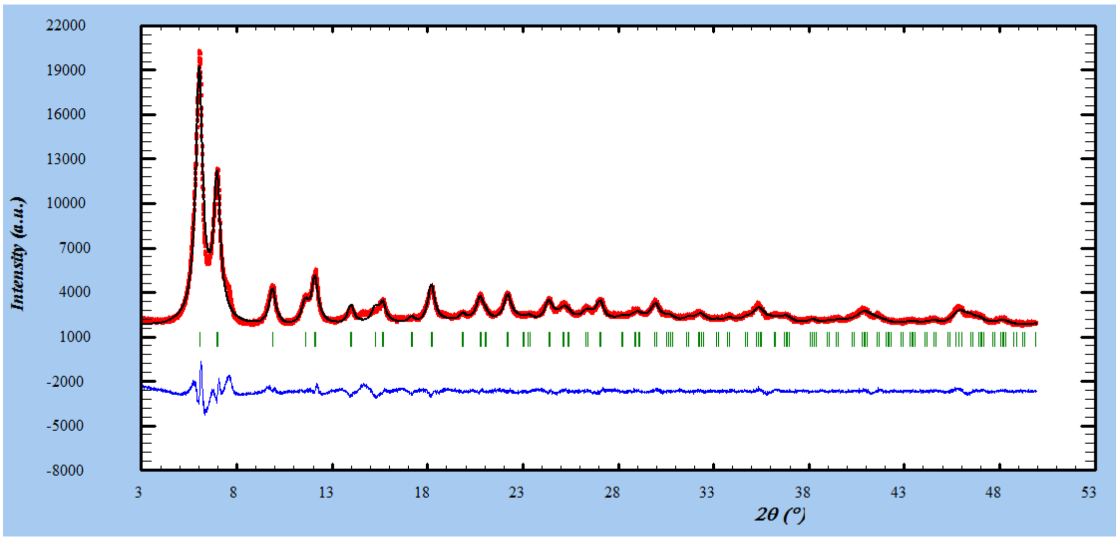

2

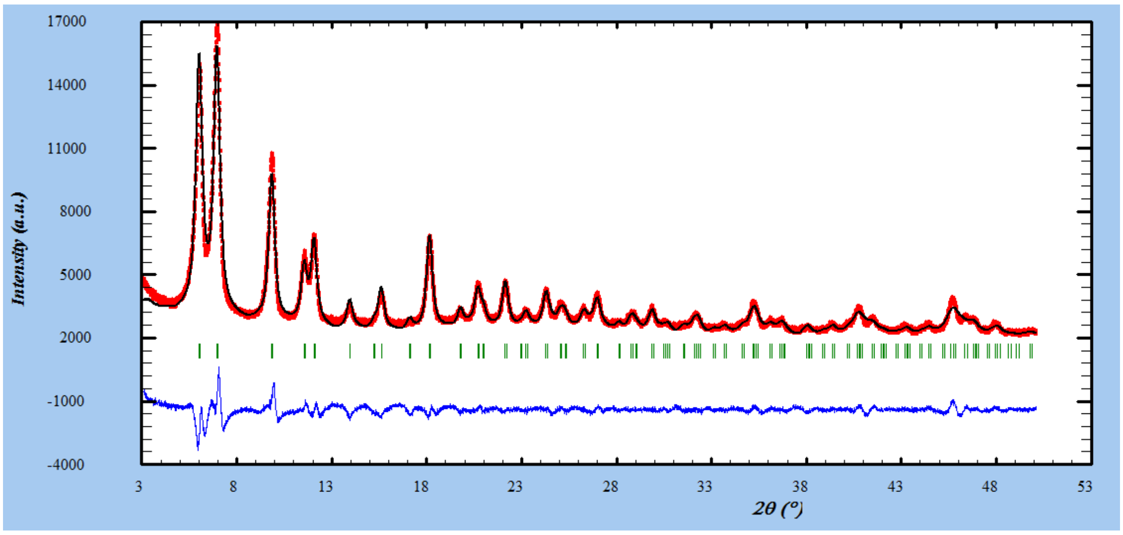

3

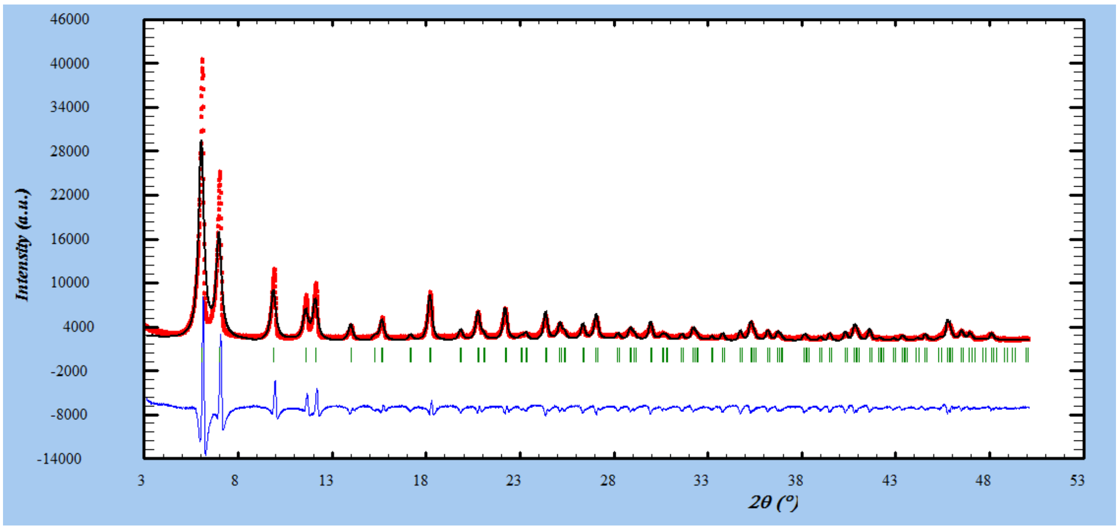

Supplementary Figure 7 / Non-structural XRPD pattern Le Bail refinements plots for 1@Ba(OH)$_2$, 2@Ba(OH)$_2$, 3@Ba(OH)$_2$, 1@KOH, 2@KOH, 3@KOH, 1, 2 and 3 (up to bottom) MOFs. Red line denote experimental data, black line denotes calculated profile, green bars denote positions calculated for Bragg reflections and blue line is the difference plot between observed and calculated profiles

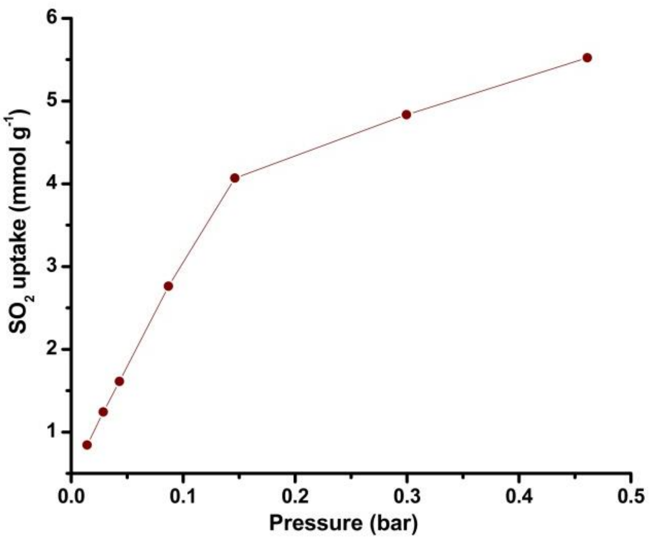

Supplementary Figure 8 / SO_2 adsorption isotherm of 1@Ba at 303K

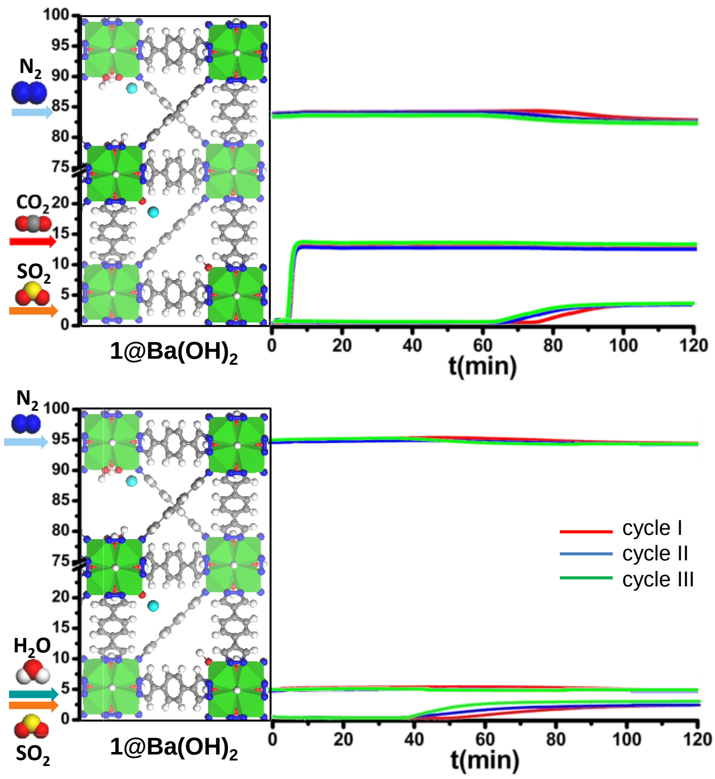

Supplementary Figure 9 / Flue gas SO_2/CO_2/N_2 (82.5:15:2.5) breakthrough curves for **1@Ba(OH)$_2$** at 303K showing the effect of the competitive adsorption of CO_2 on the capture of SO_2 (top). Effect of the presence of 80 % relative humidity on the SO_2 capture properties of **1@Ba(OH)$_2$** (down). Time scale normalized to 1g of MOF adsorbent

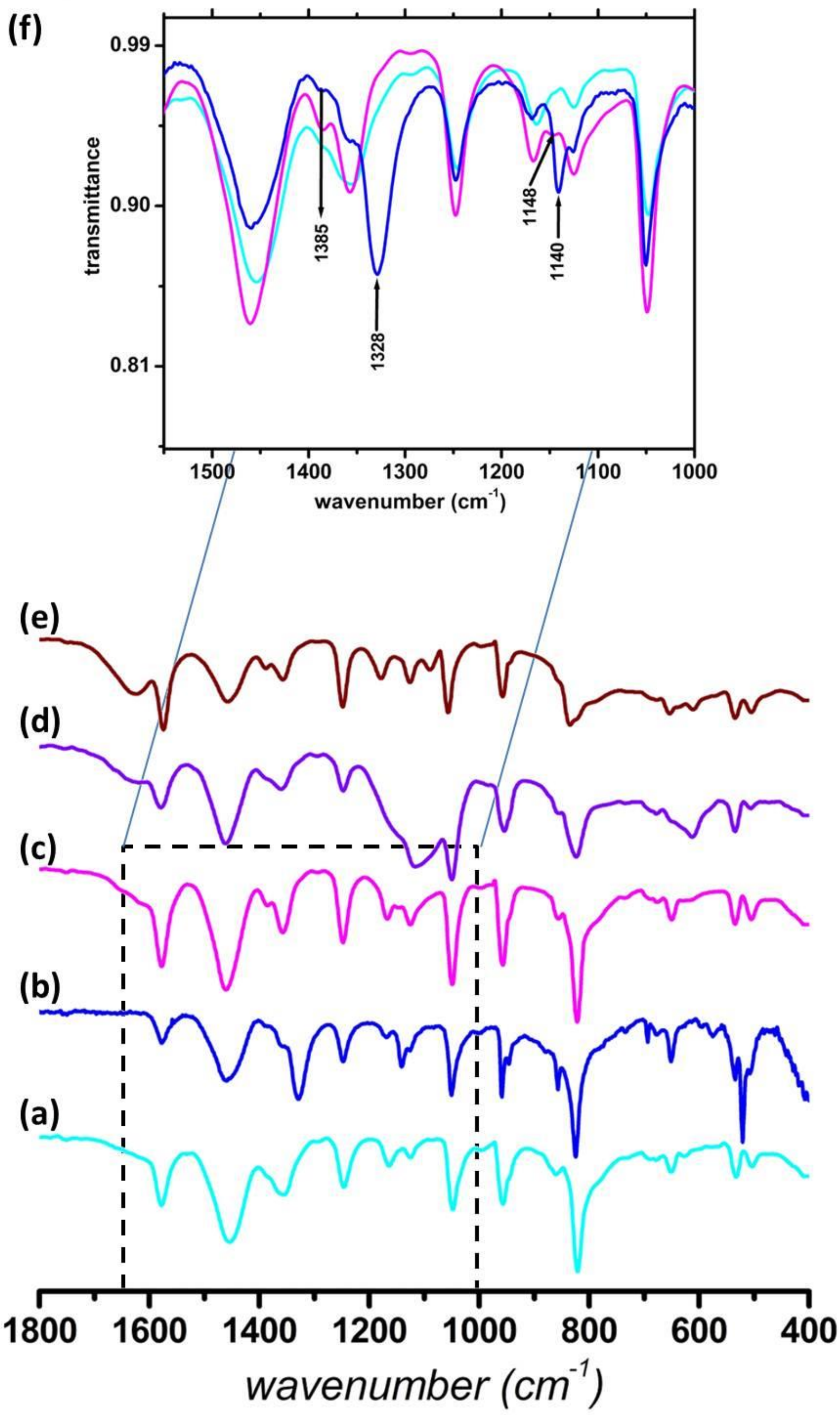

Supplementary Figure 10 / FTIR spectra comparison of **1@Ba(OH)$_2$** before(**a**), during(**b**) and after(**c**) N_2/SO_2 breakthrough experiments, (**d**) **1@Ba(OH)$_2$** after $N_2/SO_2/H_2O$ breakthrough test, (**e**) the same MOF of (**b**) under humid air flow and (**f**) zoom of spectra (**a**),(**b**) and (**c**) between 1000 to 1550 cm^{-1} . It is noticeable the occurrence of a two weak bands at 1148 and 1385 cm^{-1} that correspond to vibration bands $\gamma_{sym\ (O=S=O)}$, and $\gamma_{assym\ (O=S=O)}$ of chemically adsorbed SO_2, while 1140 and 1328 cm^{-1} correspond to symmetric and asymmetric vibration bands of physisorbed SO_2[1]

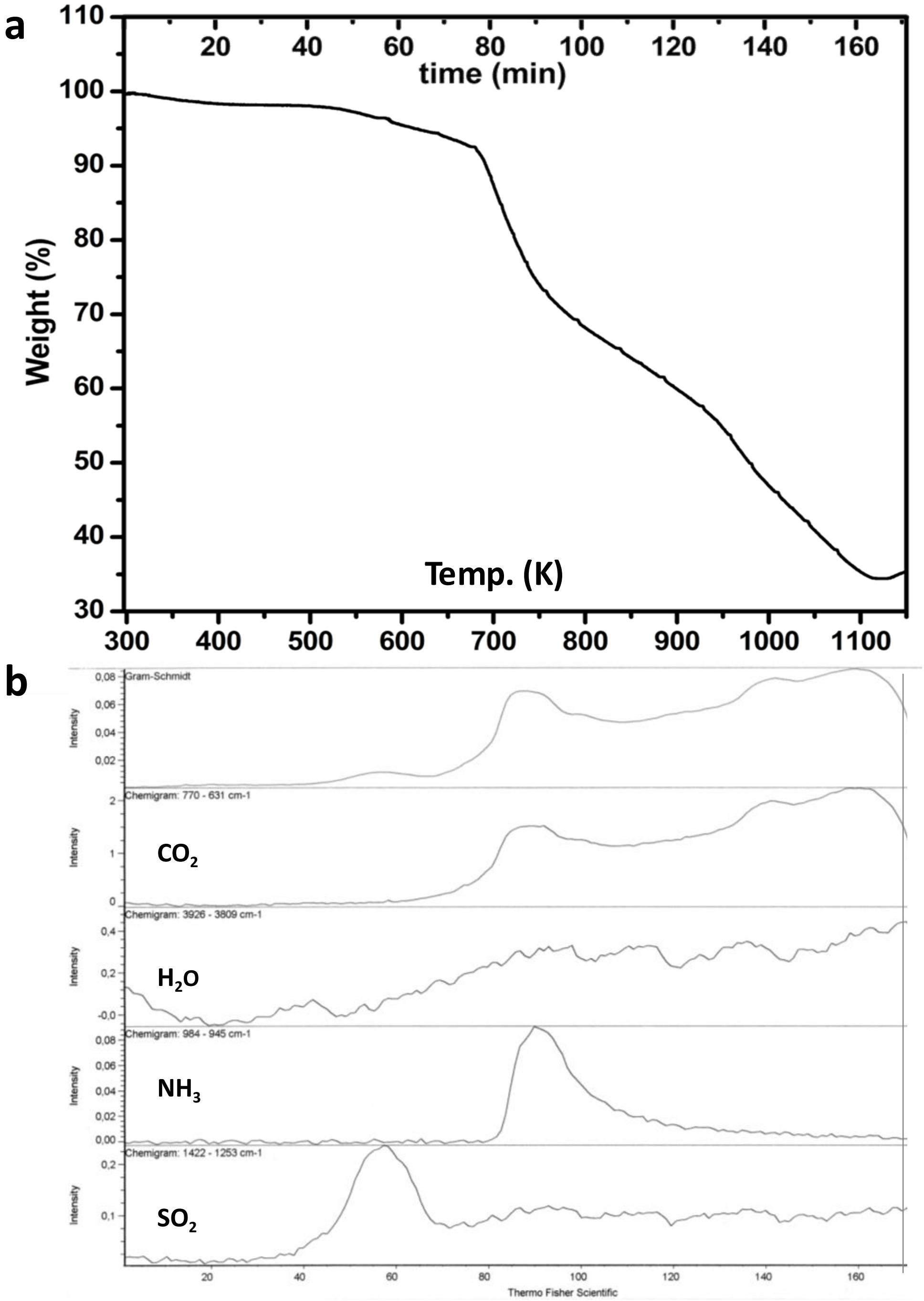

Supplementary Figure 11 / TGA-FTIR experiment of 1@Ba$(OH)_2$ after N_2/SO_2 breakthrough experiments under nitrogen atmosphere at 5K/min (a) TGA curve vs. temperature/time and (b) Gram-Schmidt diagram of all infrared active species and chemigram at 770-631cm^{-1} for CO_2, 3926-3809cm^{-1} for H_2O, 984-945 cm^{-1} for NH_3 and 1422-1253 cm^{-1} for SO_2

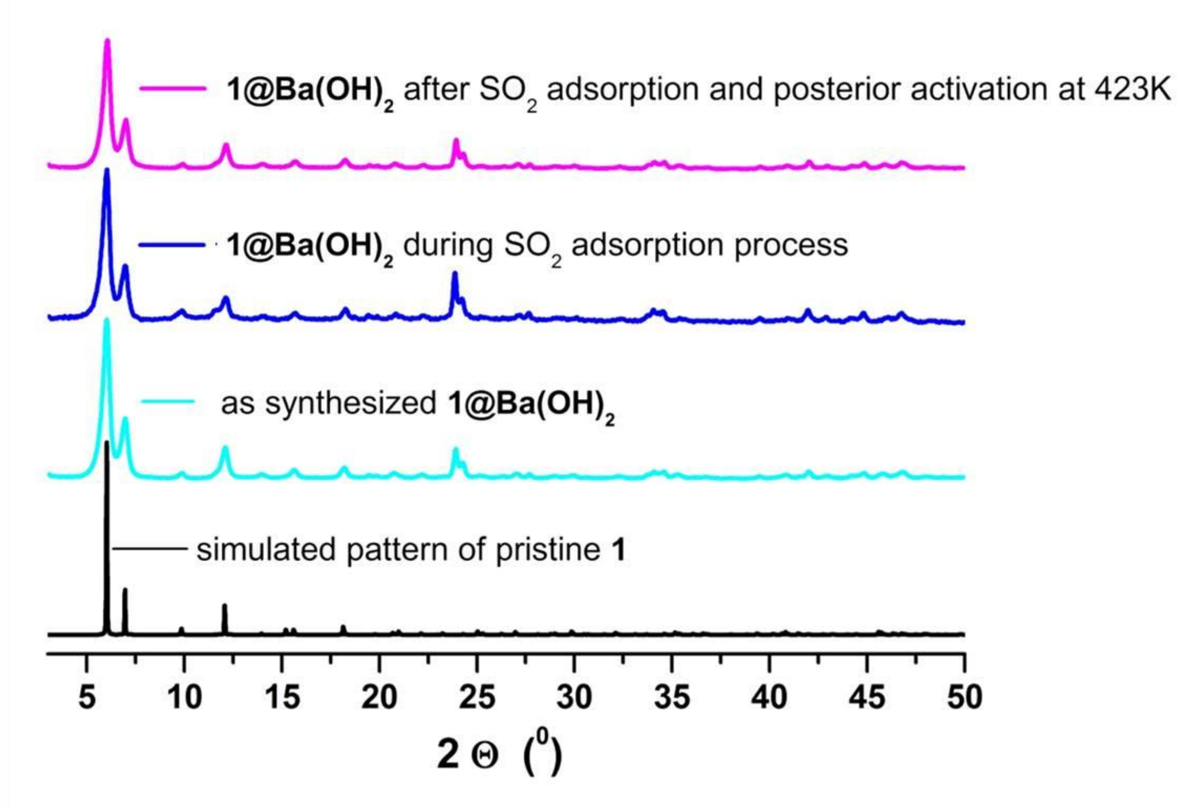

Supplementary Figure 12 / XRPD pattern of **1@Ba(OH)$_2$** before, during and after N_2/SO_2 breakthrough adsorption experiments compared to simulated pattern of pristine 1

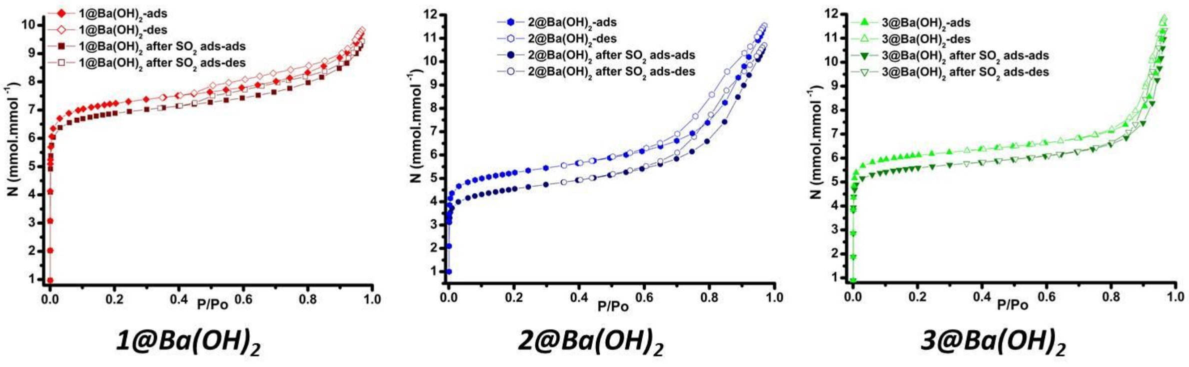

Supplementary Figure 13 / Nitrogen adsorption isotherms at 77K of **1@Ba(OH)$_2$**, **2@Ba(OH)$_2$** and **3@Ba(OH)$_2$** before and after N_2/SO_2 breakthrough experiments

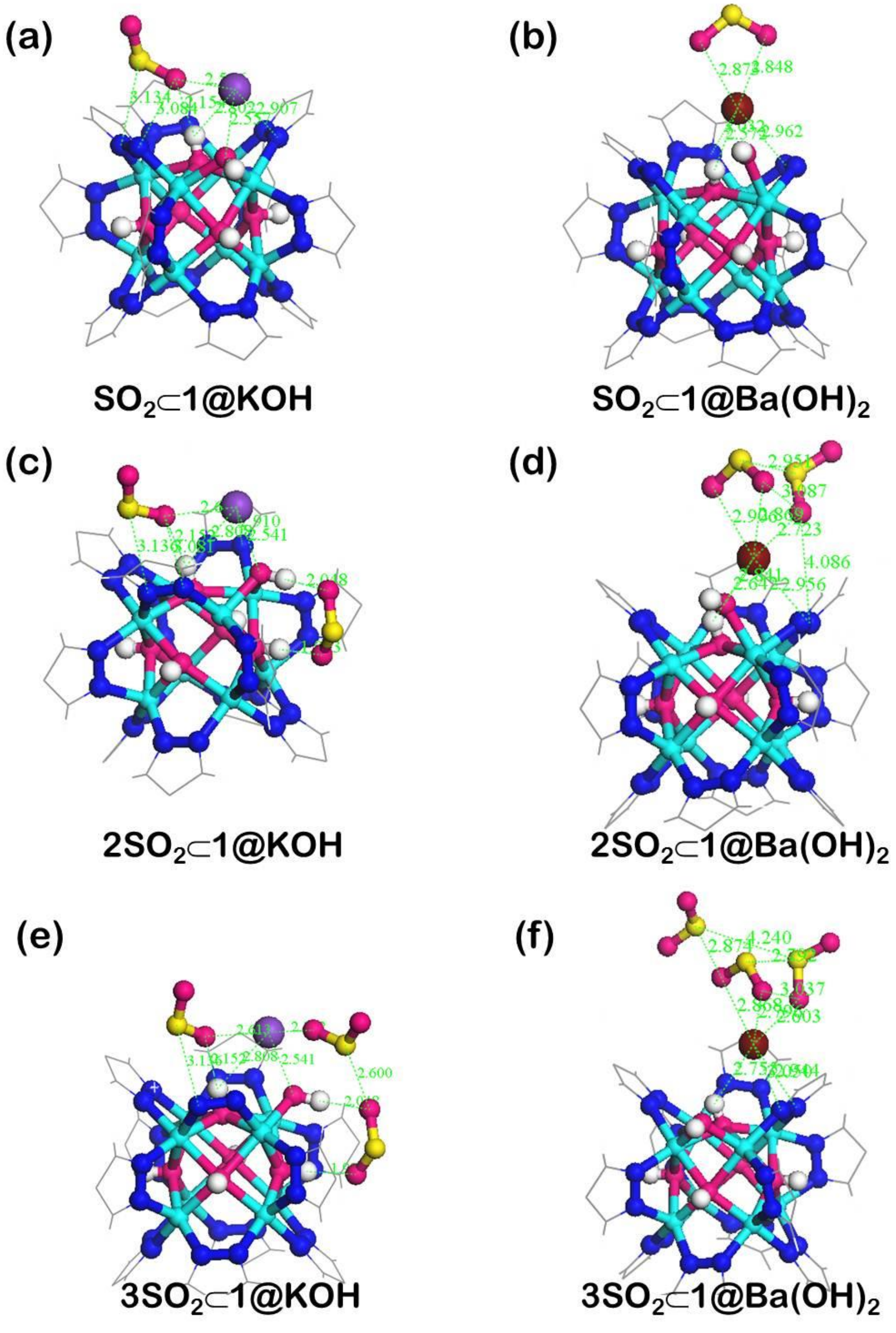

Supplementary Figure 14 / DFT energy minimised configurations of 1 (a), 2 (c) and 3 (e) SO_2 molecules adsorbed in **1@KOH**. DFT energy minimised configurations of 1 (b), 2 (d) and 3 (f) SO_2 molecules adsorbed in 1@Ba$(OH)_2$. For the sake of clarity, only the region around the metal cluster is shown. Ni (cyan); K (purple); Ba (wine); C (grey); N (blue); O (magenta); H (white); S (yellow). The binding energies are reported in Supplementary Table 7

Supplementary Table 1 / Summary of non-structure XRPD Le Bail pattern fitting results for all MOFs series compared to pattern of pristine[$Ni_8(OH)_4(H_2O)_2(C_{12}H_8N_4)_6$], Cubic Fm3m, a=25.3802Å

Cell /MOF	***1***	***2***	***3***	***1@KOH***	***2@KOH***	***3@KOH***	***1@Ba(OH)$_2$****	***2@Ba(OH)$_2$****	***3@Ba(OH)$_2$****
a (Å)	*25.31250 (0.00282)*	*25.36860 (0.00229)*	*25.29699 (0.00198)*	*25.32308 (0.00173)*	*25.38554 (0.00238)*	*25.31253 (0.00264)*	*25.39544 (0.00250)*	*25.39149 (0.00184)*	*25.34592 (0.00228)*
Volume (Å^3)	*16218.29 (3.128)*	*16326.359 (2.548)*	*16188.489 (2.195)*	*16238.645 (1.920)*	*16359.093 (2.653)*	*16218.358 (2.930)*	*16378.234 (2.794)*	*16370.601 (2.060)*	*16282.617 (2.538)*
No of ref. par.	*10*	*10*	*10*	*10*	*10*	*10*	*25*	*27*	*19*
Rp	*3.73*	*3.82*	*10.4*	*2.28*	*2.92*	*2.62*	*3.53*	*3.43*	*2.98*
Rwp	*5.94*	*5.19*	*15.4*	*2.95*	*3.95*	*3.43*	*4.63*	*4.43*	*3.90*
X^2	*7.93*	*8.96*	*77.8*	*2.58*	*3.70*	*3.23*	*3.58*	*3.38*	*3.43*

Supplementary Table 2 / Comparison of lattice cell parameters and cell volumes resulted from Le Bail profile fitting of **1@KOH**, **2@KOH, 3@KOH, 1@Ba(OH)$_2$, 2@Ba(OH)$_2$** and **3@Ba(OH)$_2$,** compared with results of corresponding as-synthesized **1**, **2** and **3**

**MOF**	**a (Å)**	**% a cell change**	**Volume (Å^3)**	**% Volume change**
**1**	25.3125		16218.29	
**2**	25.3686		16326.359	
**3**	25.29699		16188.489	
**1@KOH**	25.32308	0.042	16238.645	0.12
**2@KOH**	25.38554	0.067	16359.093	0.20
**3@KOH**	25.31253	0.061	16218.358	0.18
**1@Ba(OH)$_2$**	25.39544	0.33	16378.234	0.98
**2@Ba(OH)$_2$**	25.39149	0.09	16370.601	0.27
**3@Ba(OH)$_2$**	25.34592	0.19	16282.617	0.58

Supplementary Table 3 / SO_2 adsorption capacities of the studied MOFs. Capacities were measured over three successive cycles of breakthrough curves of a 20 mL min^{-1} flow of N_2/SO_2 (97.5:2.5) gas mixture at 303K. The values for **1@Ba(OH)$_2$-3@Ba(OH)$_2$** are calculated taking into consideration that the capacity of adsorption is related to **MOF@Ba(OH)$_2$** without the correspondent percentage of barium hydroxide

	**SO_2 adsorption (mmol g^{-1})**		
**MOF**	**Cycle I**	**Cycle II**	**Cycle III**
1	2.02	1.75	1.77
2	2.11	1.55	1.52
3	3.35	2.89	2.86
1@KOH	3.26	2.20	2.20
2@KOH	2.54	1.63	1.57
3@KOH	4.38	2.78	2.77
1@Ba(OH)$_2$	4	2.93	2.93
2@Ba(OH)$_2$	3.65	2.47	2.47
3@Ba(OH)$_2$	5.61	3.77	3.75

Supplementary Table 4 / Summary of Surface Area values for **1**-**3**, **1@KOH**-**3@KOH** and **1@Ba(OH)$_2$-3@Ba(OH)$_2$** materials and thermodynamic values of enthalpy, entropy and Gibbs enthalpy for CO_2 adsorption obtained from van't Hoff plotting of the chromatographic results

**MOF**	**SA/m^{2}g^{-1}**	**-ΔHiso/kJ mol^{-1}**	**-ΔS/J K^{-1} mol^{-1}**	**-ΔG/kJ mol^{-1}***
1	1735	26.3	108.4	-6.0
2	1385	26.4	102.1	-4.0
3	1170	30.1	109.3	-2.4
1@KOH	2055	31.8	116.7	-2.9
2@KOH	1830	33.3	112.4	-0.17
3@KOH	1665	34.0	120.5	-1.9
1@Ba(OH)$_2$	1250	33.2	126.9	-4.6
2@Ba(OH)$_2$	850	33.5	118.8	-1.9
3@Ba(OH)$_2$	1190	39.6	142.9	-3.0

*Calculated values at 298K

Supplementary Table 5 / BET surface areas before and after adsorption of SO_2 on **MOF@Ba(OH)$_2$**

**BET surface area (m^2.g^{-1})**	**1@Ba(OH)$_2$**	**2@Ba(OH)$_2$**	**3@Ba(OH)$_2$**
Before adsorption	1250	850	1190
After adsorption	1190	730	1090
differences	60	120	100

Supplementary Table 6 / Distances (Å) of bond or close contacts for pristine **1** and for **1@KOH** and **1@Ba(OH)$_2$** optimized structures

	**Ni–Ni cluster**	**Ni – cation**	**cation–OH(cluster)**	**cation–N**
**1**	3.01			
**1@KOH**	2.76 - 3.28	3.38, 3.56	2.48, 3.09	2.95-3.94
**1@Ba(OH)$_2$**	2.88 - 3.28	3.58, 4.57	2.64, 2.77	2.88-4.27

Supplementary Table 7 / Binding energies of the first, second and third H_2O and SO_2 adsorbed energies in the **1**, **1@KOH** and **1@Ba(OH)$_2$** materials. The binding energy is calculated by following equation

$$\Delta E_n = E\{F + nM\} - E\{F + (n-1)M\} - E\{M\} \qquad (1)$$

Where *F* is the Framework, *M* is the adsorbed molecule (H_2O o SO_2) and *n* is the number of adsorbed molecules. The final structures are shown in Supplementary Figure 15.

	Binding energy 1st SO_2	Binding energy 2nd SO_2	Binding energy 3rd SO_2	Binding energy 1st H_2O	Binding energy 2nd H_2O	Binding energy 3rd H_2O
1	-83.5	-78.8	-41.0	-76.3	-74.9	-69.9
1@KOH	-88.1	-65.0	-44.3	-	-	-
1@Ba(OH)$_2$	-107.4	-78.2	-42.0	-	-	-

Supplementary Note 1

We investigated the relevant siting of SO_2 by studying 6 different configurations in 1. Once these calculations were performed, three configurations were further considered for the case of 1@KOH considering variations of the local structure in connection to the missing linker. The case of SO_2 incorporation in 1@Ba(OH)$_2$ was studied with the two more stable configurations. Since it was shown above that the metal hydroxide cluster – ligand vacancy – extra-framework cation complexes are different for K^+ and Ba^{2+}. It would be expected that the interaction with the initially arriving SO_2 molecules would be different. The calculations reveal it, but surprisingly the way SO_2 interacts with the defective solids is quite different (Supplementary Figure 15 and Supplementary Table 7). While the preferential interaction of the SO_2 molecule with 1@Ba(OH)$_2$ is directly through the extra-framework cation (Ba), in the case of K the stabilization of the SO_2 molecule is produced *via* monodentate interaction with extra-framework cation (K) supplemented with a hydrogen bonding with a hydroxide cation of the metal cluster (Supplementary Figure 15 and Supplementary Table 7). These high energies are indicative of strong interactions between the framework and the adsorbed SO_2 molecules.

Supplementary Methods

All the general reagents and solvents were commercially available and used as received.

Elemental Analysis: Elemental Analysis was carried out on a Thermo Finniganon Flash EA1112 Series CHNS/O Analyzer using 2–5 mg of samples. Metal content of the samples were determined on previously acid digested and dissolved samples in aqueous solution by Inductively Coupled Plasma-Mass Spectrometry (ICP-MS) NEXION 300D instrument.

FT-IR measurements: FT-IR spectroscopy measurements were performed on a Tensor 27 Bruker Spectrometer using a Platinum Diamond crystal Attenuated Total Reflectance accessory, with 4 cm^{-1} resolution and scan range from 4000 to 400 cm^{-1}.

Thermal Gravimetric Analysis: Thermogravimetric analyses were performed, in air atmosphere, on a Shimadzu-TGA-50H equipment, at a heating rate of 20 K min^{-1}.

XRPD analysis: XRPD data were obtained on a D2 PHASER Bruker diffractometer using Cu Kα radiation (λ = 1.5418 Å) by means of a scan in the 2 θ range of 5–50° with 0.02°/1s. The compounds were manually grounded in an agate mortar and then deposited in the hollow of a zero-background silicon sample holder.

Adsorption measurements

Adsorption isotherms were measured using a Micromeritics Tristar 3000 volumetric instrument under continuous adsorption conditions. Brunauer-Emmet-Teller (BET) and Langmuir analyses were used to determine the total specific surface areas for the N_2 and CO_2 isotherms, at 77 K and 273K respectively. All the samples were activated at 423K and outgassed for 12 hours prior measurements

Electron Microscopy - Energy Dispersive X-ray diffraction measurements:

VP-SEM (Variable Pressure Scanning Electron Microscopy) analysis was performed on samples with Zeiss SUPRA40VP instrument with accelerating voltage 5 to 20kV. All the samples were measured by dispersing the material onto a sticky carbon surface attached to a flat aluminium sample holder. The samples were then, carbon coated using a BAL-TEC MED-020 Sputter at ambient temperature in inert atmosphere.

High Resolution Transmission Electron Microscopy (HRTEM). The samples were prepared as follows: materials were grounded and 1 mg was suspended in 1 mL of absolute ethanol by sonication, for 20 minutes to disperse the nanoparticles into the solution and subsequently the materials were hold using a copper Holey Carbon type grid by dipping the grid into the solution for 20 times and finally they were dried overnight before analysis. Samples were analyzed using a HAADF FEI TITAN G2 instrument with an accelerating voltage in the range 50 to 300kV and magnification up to 1,5MX.

Synthesis of ligands 4,4'-benzene-1,4-diylbis(1H-pyrazole) and derivatives

4,4'-benzene-1,4-diylbis(1H-pyrazole) The first intermediate, 1,4-bis(1-dimethylamino-3-dimethylimonio-prop-1-en-2-yl)benzene bis(perchlorate), was prepared according to methods reported [2] 44.8 mL (0.48 mol) $POCl_3$ were added drowpwise to 180 mL of DMF at 5-10 °C with constant stirring. The mixture was stirred for an additional hour at room temperature. Then 15.52 g (80.0 mmol) solid *p*-phenylenediacetic acid was added at once and the clear solution formed was stirred for 4 hours at 90-95 °C and then at room temperature overnight. The resulting black mixture was poured on 400 g crushed ice. After decomposition of the excess Vilsmeyer reagent a saturated solution of 60.0 g NaClO4 was added with stirring. The resulting nearly white crystalline deposit of the bis(trimethinium) diperchlorate was filtered and washed with two 30 mL portions of water. (35

g, yield 84 %). ^{1}H NMR (DMSO) : 2.45 (s, 3H),3.36 (s, 3H), 7.39 (s, 2H), 7.73 (s, 2H). Anal. calc. for $C_{20}H_{32}Cl_2N_4O_8$ (527.40 g/mol)

The bis(perchlorate) intermediate (4.70 g, 8.9 mmol) in EtOH (600 mL) was placed in a 1 L round bottomed flask under vigorous stirring, while 98% hydrazine monohydrate (90.5 µL, 19.6 mmol) was added dropwise over 2 min. After being refluxed for 2 h, the reaction was stirred overnight at room temperature. The pale-yellow solid was filtered off, washed with ethanol (2 × 100 mL) and dried under vacuum at room temperature to afford pure ligand as polycrystalline powder ((1.8 g, yield 99 %).. ^{1}H NMR (DMSO): 7.58 (s, 2H), 8.05 (s, 2H), 12.5 (br s, 1H). 13C. Anal. calc. for $C_{12}H_{10}N_4$ (210.24 g/mol.

For the preparation of derivatives amino- and hydroxyl-4,4'-benzene-1,4-diylbis(1H-pyrazole) the following methods were used:

2-Nitro[1,4-bis(1H-pyrazol-4-yl)benzene] (H_2BDP_NO_2). 1,4-Bis(1H-pyrazol-4-yl)benzene (1.000 g, 4.76 mmol) was added in portions to concentrated sulphuric acid (10 mL) while keeping the reaction mixture cold with an ice bath. To the solution was then added 70% nitric acid (0.255 mL, 5.71 mmol) dropwise while maintaining the reaction mixture cold. The ice bath was then removed, and the solution was left at room temperature under stirring for 1 h. Next, 10 g of crushed ice was added, and the precipitate was filtered off and washed with 10 mL (2 × 5 mL) of water. The precipitate was neutralized with aqueous NaHCO3, and the resulting product was collected by filtration and washed with 10 mL of water (2 × 5 mL), affording the pure ligand as a yellow solid (1.23 g, yield 98%).. ^{1}H NMR (DMSO-d6): δ 7.63 (d, 1H), 7.79 (s, 2H), 7.89 (dd, 1H), 8.08 (d,1H), 8.23 (s, 2H). Anal. Calc. for $C_{12}H_9N_5O_2$ (MW = 255.2 g/mol).

2-Amino[1,4-bis(1H-pyrazol-4-yl)benzene] (H_2BDP_NH_2). To a suspension of H_2BDP_NO_2 (0.9 g, 3.528 mmol) in DMF (15 mL) was added ammonium formate (1.11 g, 17.619 mmol) at room temperature. The reaction mixture was then heated to 120 °C, and Pd/C (5%, 90 mg) was added in small portions. The final mixture was then kept under stirring at 100 °C for 2 h. After the reaction mixture became clear, it was filtered through Celite pad. The Celite pad was then washed with a small amount of DMF, and the filtrate was diluted with crushed ice (30 g). The obtained precipitate was then filtered off and washed with water (2 × 15 mL), affording a white powder of pure H_2BDP_NH_2 product (0.75 mg, yield 96%). 1H NMR (DMSO-d6): δ 4.77 (s, 2H), 6.84 (dd, 1H), 6.97 (d, 1H), 7.15 (d, 1H), 7.86 (br s, 4H), 12.88 (br s, 2H). Anal. Calc. for $C_{12}H_{11}N_5$ (MW = 225.2 g/mol.

2-Hydroxo[1,4-bis(1H-pyrazol-4-yl)benzene] (H_2BDP_OH). One gram (4.444 mmol) of H_2BDP_NH_2 was dissolved in 5 mL of sulfuric acid. The mixture was stirred until a thick paste was formed. To this was added about 3 g of crushed ice, and the mixture was then kept in an ice bath. In a separate beaker, $NaNO_2$ (0.440 g, 5.176 mmol) was dissolved in 4 mL of water. This solution was cooled and added dropwise, with constant stirring, to the acid amine solution. In a separate flask, a solution of H_2SO_4 (3 mL) and water (3 mL) was heated to 110 °C, and the entire diazonium salt solution was added dropwise. After the addition was over, the solution was allowed to boil for another 30 min. It was then cooled with an ice bath, and the precipitate was filtered off and suspended in a solution of $NaHCO_3$ in water and stirred for 2 h at 80 °C. The yellowish precipitate was then filtered off, washed with water (2 × 5 mL), and dried under vacuum (0.9 g, yield 90%). ^{1}H NMR (DMSO-d6): δ 7.04 (m, 2H), 7.51 (d, 1H), 8.04 (br s, 4H), 9.67 (s, 1H), 12.83 (br s, 2HAnal. Calc. for $C_{12}H_{10}N_{4O}$ (MW = 226.2 g/ mol.

*Synthesis of materials **1, 2** and **3***

The synthesis of MOFs samples 1-3 were prepared according to the procedure reported by our group[3], with subtle changes as follows: in a typical synthesis, 631mg (3mmol) of 4,4'-benzene-1,4-diylbis(1H-pyrazole) were dissolved in 160 mL of N,N'-dimethylformamide and 992 mg (4mmol) of $Ni(CH_3COO)_2$ 4 H_2O were dissolved in 40 mL of H_2O. The two solutions were mixed and refluxed for 12 h under stirring. The solid obtained was filtered off and washed with N,N'-dimethylformamide, ethanol and diethyl ether, yielding the corresponding MOF ***1-3***. Prior to use or characterization of materials, 500mg of as-synthesized solids were solvent exchanged with 100ml of dichloromethane, with stirring at room temperature for 2 h.

Preparation of materials ***1@KOH,2@KOH*** *and* ***3@KOH***

The postsynthetical modification of **1** , **2** and **3** materials were done according to previously reported procedure by our group[4], with activation of as synthesized MOFs thermally at 423 K and outgassed to 10 $^{-1}$ Pa for 12h, in order to obtain solvent-free porous matrix. Afterwards, 0.055 mmol of each material was suspended in 0.35 M KOH absolute ethanol solution (5.5 mL). The resulting suspensions were stirred overnight under an inert N_2 atmosphere, filtered off and washed copiously with absolute ethanol yielding the corresponding compounds **1@KOH**, **2@KOH**, **3@KOH**.

Preparation of materials $Ba_{0.5}(Ni_8(OH)_3(C_2H_5O)_3(H_2O)_2(C_{12}H_8N_4)_{5.5})(Ba(OH)_2)(H_2O)_5$ (**1@Ba(OH)$_2$**), $Ba_{1.5}(Ni_8(OH)_3(C_2H_5O)(H_2O)_2(C_{12}H_7N_4O)_5)(Ba(OH)_2)_{0.5}(H_2O)$, (**2@Ba(OH)$_2$**) and $Ba_{0.5}(Ni_8(OH)_3(C_2H_5O)_3(H_2O)_2(C_{12}H_9N_5)_{5.5})(Ba(OH)_2)_{1.5}(H_2O)$ (**3@Ba(OH)$_2$**)

The materials **1@KOH** , **2@KOH** , **3@KOH** were used as prepared without previous activation as follow, 100 mg of the **1@KOH-3@KOH** materials were suspended in 12 mL of a 0.1M aqueous solution of the $Ba(NO_3)$ with stirring for 72h at room temperature. The postmodified materials were subsequently filtered off, washed with water and ethanol and dried in air. Later, the solids (~50mg) as obtained were suspended in 50ml of water for 4 hours in order to remove the eventual absorbed ion pairs. The materials **1@Ba(OH)$_2$, 2@Ba(OH)$_2$** and **3@Ba(OH)$_2$** were filtered off and washed with water and ethanol, and dried in air.

Postsynthethical modified material **1@Ba(OH)$_2$:** Elemental analysis (previously activated sample) Calculated for $Ba_{0.5}(Ni_8(OH)_3(C_2H_5O)_3(H_2O)_2(C_{12}H_8N_4)_{5.5})(Ba(OH)_2)(H_2O)_5$.% C,39.9; N, 14.22; H, 3.63; Found, C,40.05; N,14.32; H, 3.90. Calculated residual oxides from TGA for **1@Ba(OH)$_2$** : $(NiO)_8(BaO)_{1.5}$ 38.18%; Found: 38.26%. ICP-MS composition for **1@Ba(OH)$_2$**: Ni, 5.75ppm; Ba, 2.82ppm. ATR-FTIR (4000 – 400 cm^{-1}) : 3585(br), 3373(br), 3141(br), 3023(w), 2967(w), 1578(s), 1453(vs), 1356(m), 1246(m), 1164(w), 1123(w), 1048(s), 956(s), 820(vs), 651(w),532(w),503(w).

Postsynthethical modified material **2@Ba(OH)$_2$:** Elemental analysis (previously activated sample) Calculated for $Ba_{1.5}(Ni_8(OH)_3(C_2H_5O)(H_2O)_2(C_{12}H_7N_4O)_5)(Ba(OH)_2)_{0.5}(H_2O)$.% C,36.73; N, 13.81; H, 2.48; Found, C,36.88; N,13.46; H, 3.05. Calculated residual oxides from TGA for **2@Ba(OH)$_2$** : $(NiO)_8(BaO)_2$ 44.5%; Found: 43.83%. ICP-MS composition for **2@Ba(OH)$_2$**: Ni, 6.90ppm; Ba, 4.10ppm. ATR-FTIR (4000 – 400 cm^{-1}) : 3388(br), 3064(w), 2975(w), 1623(m), 1573(s), 1456(vs), 1371(m), 1249(m), 1211(w), 1170(w), 1058(s), 958(m), 856(vs), 823(s), 692(w), 665(w),613(w),567(w).

Postsynthethical modified material **3@Ba(OH)$_2$:** Elemental analysis (previously activated sample) Calculated for $Ba_{0.5}(Ni_8(OH)_3(C_2H_5O)_3(H_2O)_2(C_{12}H_9N_5)_{5.5})(Ba(OH)_2)_{1.5}(H_2O)$.% C,38.20; N, 17.02; H, 3.41; Found, C,38.11; N,17.02; H, 3.83. Calculated residual oxides from TGA for **3@Ba(OH)$_2$** : $(NiO)_8(BaO)_2$

39.95%; Found: 40.15%. ICP-MS composition for **3@Ba(OH)$_2$**: Ni, 6.11ppm; Ba, 3.29ppm. ATR-FTIR (4000 – 400 cm^{-1}) : 3583(w), 3365(br), 3045(w), 2970(w), 1622(m), 1572(s), 1460(vs), 1384(m), 1246(s), 1168(m), 1126(w), 1058(vs), 958(m), 856(m), 810(vs), 665(w), 617(w), 563(w), 501(w), 474(w).

Breakthrough and Pulse Gas Chromatography Experiments

Adsorption isotherm of SO_2 on 1@Ba at 303K

Adsorption isotherm was measured at 303K, point by point using breakthrough experiments with total flow of 30 mL min^{-1} He/SO_2 variable gas mixtures from (97.5/2.5) to (25/75). The MOFs is activated at 423K for 24 hours before first chemisorption cycle, and for 2 hours between further cycles. Each gas mixture is measured twice in order to assure the physisorption process. The adsorbed amount was calculated using the same procedure as for routine breakthrough experiments. It should be noted that all isotherm points are measured on the same prepared column and the material characterized after this adsorption cycles maintain crystallinity and porosity. The desorption branch was unable to measure with this procedure.

Breakthrough Experiments for Gas Separation

For these measurements, the PSM materials used were carefully handled avoiding possible chemisorption of CO_2 from air and the 20-cm chromatographic column, that was prepared employing a stainless steel 20 cm-column (0.4 cm internal diameter) packed with ca. 0.5 g of the studied materials (**1**-**3**, **1@KOH**-**3@KOH** and **1@Ba(OH)$_2$**-**3@Ba(OH)$_2$**. The column was activated under a pure He flow (20 mL min^{-1}) at 423 K overnight and for two hours between successive breakthrough cycles. The desired gas mixture (20 mL min^{-1}) was prepared via mass flow controllers. For instance, N_2/SO_2 (97.5 : 2.5), $N_2/CO_2/SO_2$ (83.5 : 14 : 2.5), and $N_2/H_2O/SO_2$ (94.1 : 3.4 : 2.5) gas mixtures were prepared in order to simulate the emission of flue gas from a power plant. The breakthrough experiments were carried out, at 303 K, by step changes from He to N_2/SO_2, $N_2/CO_2/SO_2$, and $N_2/H_2O/SO_2$ flow mixtures. The subsequent breakthrough cycles were measured with prior sample reactivation under a pure He flow (20 mL min^{-1}) at 423 K during 2 h. The relative amounts of gases passing through the column were monitored on a Mass Spectrometer Gas Analysis System (Pfeiffer Vacoon) detecting ion peaks at m/z 64 (SO_2), 44 (CO_2), 28 (N_2), 18 (H_2O) and 4 (He). The adsorbed amounts of SO_2 for the different materials are summarized in Supplementary Table 3.

Variable temperature pulse gas chromatography

Gas-phase adsorption at zero coverage surface was studied using the pulse chromatographic technique[5] employing a gas chromatograph and stainless steel 20 cm-column (0.4 cm internal diameter) packed with ca. 0.5 g of the studied materials (**1**-**3**, **1@KOH**-**3@KOH** and **1@Ba(OH)$_2$**-**3@Ba(OH)$_2$**). It should be noted that all the measurements were done on the materials after SO_2 chemisorption (after breakthrough experiments) in order to ensure the thermodynamic equilibrium. Prior to measurement, samples were heated overnight at 423 K in a He flow (30 mL min^{-1}). Later on, an equimolecular gas mixture composed of H_2, N_2, CO_2, SO_2 gases (0.4 mL) was injected at 1 bar and the separation performance of the chromatographic column was examined at different temperatures (403 K-433 K) by means of a mass Spectrometer Gas Analysis System (Pfeiffer Vacoon), detecting the corresponding masses. The dead volume of the system was calculated using the retention time of hydrogen as a reference. The zero-coverage thermodynamic parameters of the

adsorption process for SO_2 and CO_2 are gathered in Tables 1 and S4, respectively. These values were calculated using a van't Hoff type analysis employing isothermal chromatographic measurements.[Error! Bookmark not defined.] The retention volumes were corrected taking into account the volume expansion of the gas entering the capillary due to the temperature increase according to $V_S = (t_R - t_m)F_a(T/T_a)j$ where V_S = net retention volume (mL); t_R = retention time (min); t_m = dead time (min); F_a = volumetric flow-rate measures at ambient temperature (ml min^{-1}); T= column temperature (K); T_a = ambient temperature (K); the James–Martin gas compressibility correction $j= (3(p_i/p_0)^2-1)/(2(p_i/p_0)^3-1)$ where p_i = pressure of gas applied to the chromatogram and p_0 = pressure of gas at outlet.

Once these corrections where applied, the van't Hoff plot of the equation $\ln V_S= \ln(RTn_s) + \Delta S/R - \Delta H_{diff}/(RT)$ was used to calculate the thermodynamic parameters of each analyte taking into account that the term $\ln(RTn_s)$ is usually small and can be neglected in the determination of ΔS. In addition to the ΔH_{diff} value obtained from the van't Hoff plot the isosteric heat of adsorption (ΔH_{iso}) was also determined according to the relation $|\Delta H_{iso}| = |\Delta H_{diff}| + RT_{average}$. The $\alpha_{SO2/CO2}$ partition coefficients have been calculated from the henry constants ratio

Characterization of materials after SO_2 chemisorption

The materials were characterised after SO_2 chemisorption process by means of EA, XRPD, FTIR, TEM-EDX, TGA-FTIR and N_2 adsorption isotherms in order to know the effect of the chemisorption process on the structural integrity of the material. The results are indicative that the crystal phase of both MOF and $Ba(OH)_2$ cocrystals are maintained and only a slight diminution on surface area is observed as a probable consequence of the formation of $BaSO_3$ nanoclusters.

*Elemental analysis of samples of **1-3**, **1@KOH-3@KOH** and **1@Ba(OH)$_2$-3@Ba(OH)$_2$** after adsorption of SO_2.*

1@Ba(OH)$_2$ after adsorption of SO_2 **:** Elemental analysis : Found % C,36.81; N,13.45; H, 3.56; S, 0.29. Calculated for $Ba_{0.5}(Ni_8(OH)_4(C_2H_5O)_2(C_{12}H_8N_4)_{5.5})(Ba(OH)_2)(H_2O)_{14}(SO_2)_{0.2}$ C,36.9; N,13.52; H, 3.89; S, 0.28. ATR-FTIR (4000 – 400 cm^{-1}) : 3360(br), 3028(w), 1578(vs), 1464(vs), 1385(w), 1355(s), 1247(vs), 1166(m), 1145(w), 1124(m), 1047(s), 955(s), 822(vs), 857(w), 651(w), 677(w), 649(m), 534(m),503(m).

2@Ba(OH)$_2$ after adsorption of SO_2**:** Elemental analysis: Found C,29.72; N, 10.91; H, 3.51; S, 0.59; calculated for $Ba_{1.5}(Ni_8(OH)_3(C_2H_5O)(H_2O)_2(C_{12}H_7N_4O)_5)(Ba(OH)_2)_{0.5}(H_2O)_{27}(SO_2)_{0.5}$ C,29.46; N, 11.08; H, 4.08; S, 0.63. ATR-FTIR (4000 – 400 cm^{-1}) : 3385(br), 1625(m), 1578(s), 1459(vs), 1375(w), 1249(m), 1201(w), 1188(w), 1088(w), 1057(s), 957(w), 943(w), 856(s), 814(s), 693(w), 673(w), 611(m),571(w), 557(w).

3@Ba(OH)$_2$ after adsorption of SO_2**:** Elemental analysis : Found: C, 33.46; N, 14.14; H, 3.42; S, 0.46; Calculated for $Ba_{0.5}(Ni_8(OH)(C_2H_5O)_5(H_2O)_2(C_{12}H_9N_5)_{5.5})(Ba(OH)_2)_{1.5}(H_2O)_{20}(SO_2)_{0.4}$ C, 33.96; N,14.33; H, 4.59; S, 0.47. ATR-FTIR (4000 – 400 cm^{-1}) : 3580(w), 3380(br), 1624(m), 1573(s), 1454(vs), 1386(m), 1373(w), 1246(s), 1172(m), 1122(w), 1108(w), 1060(vs), 985(m), 946(w), 856(s), 822(s), 693(w), 668(w), 613(s), 571(w)according to the relation the $\alpha_{SO2/CO2} = K_{H\_SO2}/K_{H\_CO2}= \exp[-(\Delta G_{SO2}-\Delta G_{CO2})/RT]$.

X-ray Crystallography for Le Bail profile fitting

X-ray powder diffraction patterns of activated samples were collected at Phillips Analytical B.V. Instrument at ambient temperature and pressure, in reflectance Bragg-Brentano geometry employing Ni filtered CuKα lines focused radiation (1.54059 Å, 1.54439 Å) at 45kV power and 40mA current, 1/4°divergence slit, 1/2° antiscatter slit. For Le Bail fitting the patterns of all samples were collected. The Le Bail fitting was carried out using Fullprof software package via the Winplotr interface[6]. To define the pattern profile, a triple Pseudo-Voight function was employed. For background file a 4-coefficient expression correction was used. The instrumental parameters were determined by refining a profile from a standard calcite sample. The following parameters have been allowed to refine: a lattice cell parameter, zero shift and profile parameters.

For the patterns of **1@Ba(OH)$_2$, 2@Ba(OH)$_2$** and **3@Ba(OH)$_2$** it was necessary to fit with three different phases of Barium hydroxide, the crystalline phases were:

- $Ba(OH)_2.8H_2O$, Monoclinic *A 2/a*, a=11.845 Å, b=9.277 Å, c=9.292 Å, β=9896
- $Ba(OH)_2.3H_2O$, Orthorhombic *Pnna*, a=7.64 Å, b=11.4 Å, c=5.966 Å
- $Ba(OH)_2$. H_2O, Monoclinic *P21/n,* a=7.049 Å, b=4.184 Å, c=6.33 Å, β=111.45

Computational details of periodic DFT calculations. Study of the adsorption of SO$_2$ molecules on defective MOFs

A theoretical study of **1, 1@KOH**, and **1@Ba(OH)$_2$** was carried out using density functional theory, as implemented in the VASP program[7]. The calculations were performed with a cut-off energy of 500 eV and PAW potentials.[8] The PBE exchange-correlation functional[9], with corrected van der Waals interactions introduced via the D2 Grimme scheme[10] was used. Due to the large sizes of the unit cells, only the gamma point was used. No symmetry constrains were used and both the atomic coordinates and the cell parameters were allowed to vary.

Calculations on **1, 1@KOH, 1@Ba(OH)$_2$** were performed with a cubic primitive cell containing 166 and 306 framework atoms for **1,** and for **1@KOH** and **1@Ba(OH)$_2$** respectively. The initial cubic cell has the cell parameters equal to *a* = 25.38 Å. Due to the presence of local defects, the optimized cells depart from the ideally cubic structure, as it is common in defective porous materials.[11] This is not a problem in the present study, as our interest lies on the local host-guest interactions. What it is important is the accurate description of the local structure and the host-guest interactions. Note that indeed both bond lengths and angles are in agreement with reported crystal data.

Computational study of the adsorption of SO$_2$ molecules

The initially high symmetry of the material allows us to model the defective solid with a relative small number of configurations. In this context, three different configurations were considered for the extraction of the missing linkers and the introduction of K^+ cations. The dangling metal-N bonds were capped with OH. In the case of the replacement of two K^+ cations by one Ba^{2+} one, four configurations were taken into account. As mentioned above, the local structure is strongly affected by the presence of defects, as can be seen in Supplementary Figure 14 and Supplementary Table 6. It is observed that larger distortions result from the exchange of K^+ by Ba^{2+} cations, as a consequence of the two times larger polarizing power of Ba^{2+}.

Supplementary References

[1] Tan, Kui, et al. "Competitive Coadsorption of CO2 with H2O, NH3, SO2, NO, NO2, N2, O2, and CH4 in M-MOF-74 (M= Mg, Co, Ni): The Role of Hydrogen Bonding." *Chemistry of Materials* 27.6: 2203-2217, (**2015**)

[2] Lozan, V. et al. "Tetranuclear Nickel Complexes Composed of Pairs of Dinuclear LNi2 Fragments Linked by 4, 4′-Bipyrazolyl, 1, 4-Bis (4′-pyrazolyl) benzene, and 4, 4′-Bipyridazine: Synthesis, Structures, and Magnetic Properties." *European Journal of Inorganic Chemistry*. 20, 3217-3226 (**2007**)

[3] Quartapelle Procopio, Elsa, et al. "Cation-Exchange Porosity Tuning in Anionic Metal–Organic Frameworks for the Selective Separation of Gases and Vapors and for Catalysis." *Angewandte Chemie* 122.40, 7466-7469. (**2010**)

[4] López-Maya, Elena, et al. "Improved CO2 Capture from Flue Gas by Basic Sites, Charge Gradients, and Missing Linker Defects on Nickel Face Cubic Centered MOFs." *Advanced Functional Materials* 24.39: 6130-6135, (**2014**)

[5] Münch, A. S. and Mertens, F. O. R. L. HKUST-1 as an open metal site gas chromatographic stationary phase—capillary preparation, separation of small hydrocarbons and electron donating compounds, determination of thermodynamic data, J. Mater. Chem., 22, 10228 (**2012**)

[6] T. Roisnel, J. Rodríquez-Carvajal, "WinPLOTR: A Windows Tool for Powder Diffraction Pattern Analysis", Materials Science Forum, Vols. 378-381, pp. 118-123, (**2001)**

[7] Kresse, Georg, and Jürgen Furthmüller. "Efficient iterative schemes for ab initio total-energy calculations using a plane-wave basis set." *Physical Review B* 54.16 11169 (**1996**):.

[8] Kresse, G., & Joubert, D. From ultrasoft pseudopotentials to the projector augmented-wave method. *Physical Review B*, *59*(3), 1758, (**1999**).

[9] Perdew, J. P., Burke, K., & Ernzerhof, M. Generalized gradient approximation made simple. *Physical review letters*,*77*(18), 3865, (**1996**)

[10] Grimme, S.. Semiempirical GGA-type density functional constructed with a long-range dispersion correction. *Journal of computational chemistry*,*27*(15), 1787-1799, (**2006**)

[11] Ruiz-Salvador, A. R., Almora-Barrios, N., Gómez, A., & Lewis, D. W. Interplay of water, extra-framework cations and framework atoms in the structure of low-silica zeolites: the case of the natural zeolite Goosecreekite as studied by computer simulation. *Physical Chemistry Chemical Physics*, *9*(4), 521-532. (**2007**).
